# Targeting chaperon protein HSP70 as a novel therapeutic strategy for FLT3-ITD-positive acute myeloid leukemia

**DOI:** 10.1038/s41392-021-00672-7

**Published:** 2021-09-15

**Authors:** Chen Hu, Fengming Zou, Aoli Wang, Weili Miao, Qianmao Liang, Ellen L. Weisberg, Yinsheng Wang, Jing Liu, Wenchao Wang, Qingsong Liu

**Affiliations:** 1 Anhui Province Key Laboratory of Medical Physics and Technology, Institute of Health and Medical Technology, Hefei Institutes of Physical Science, Chinese Academy of Sciences, Hefei, Anhui People’s Republic of China; 2 Hefei Cancer Hospital, Chinese Academy of Sciences, Hefei, Anhui People’s Republic of China; 3 Department of Chemistry, University of California-Riverside, Riverside, CA USA; 4 University of Science and Technology of China, Hefei, Anhui People’s Republic of China; 5 Department of Medical Oncology, Dana Farber Cancer Institute, Harvard Medical School, Boston, MA USA

**Keywords:** Drug development, Target validation

**Dear Editor**,

Approximately 25% of acute myeloid leukemia (AML) carries FLT3-ITD (internal tandem duplication) oncogenic mutations. Although FLT3 kinase inhibitors have already been successfully used in the clinic for treating FLT3-ITD-positive AML, acquired drug resistance is observed after the prolonged treatment. Therefore, seeking a new therapeutic strategy is still imperative for FLT3-ITD-positive AML.

During the study of kinase inhibitors against AML, we found that the BTK inhibitor QL-XII-47 (QL47; Supplementary Fig. S1a) exhibited potent antiproliferative activity against AML cell lines (MOLM13/MOLM14/MV-4-11) carrying FLT3-ITD mutations, while two other BTK inhibitors had no obvious effect (Fig. 1a). Moreover, as we reported previously, BTK knockdown had no effect on MOLM13 proliferation^1^. In addition, kinase profiling confirmed that QL47 did not bind FLT3-WT or FLT3-ITD^2^. Therefore, we excluded the possibility that QL47 mediates its inhibitory activity through BTK or FLT3.

**Fig. 1:**
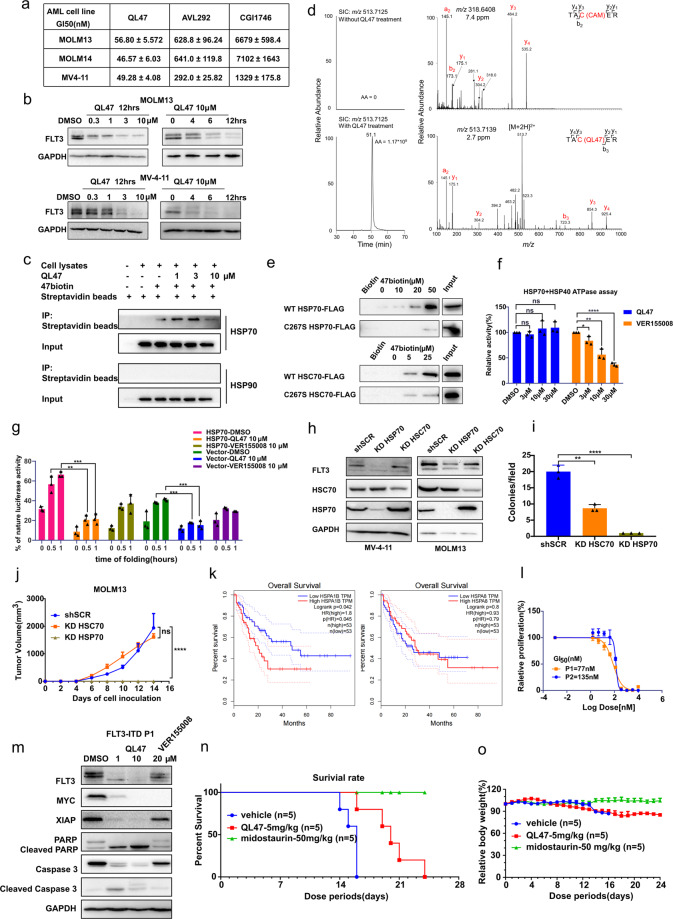
**a** For 72 h treatment, QL47 inhibited FLT3-ITD-positive AML proliferation more potently compared to BTK inhibitor AVL292 and CGI1746 measured by CellTiter-Glo assay. **b** QL47 induced FLT3-ITD decrease in a dose-and time-dependent manner. **c** QL47 preincubated MV-4-11 cell lysis were exposed to 47biotin for 4 h, then precipitated by streptavidin agarose. After PBS washing, heat shock proteins binding with 47biotin were detected by western blotting, using HSP70 and HSP90 antibodies. **d** LC-MS and MS/MS for the identification of QL47 modification site in HSP70. Shown on the left are the selected-ion chromatograms (SICs) for monitoring the [M + 2H]^2+^ ion (*m*/*z* 513.7125) of the tryptic peptide TACER with Cys267 being modified by QL47 in the tryptic digestion mixture of HSP70 without (top) or with (bottom) QL47 treatment. Displayed on the right are the MS/MS for the [M + 2H]^2+^ ions of the tryptic peptide TACER with Cys267 being carbamidomethylated (CAM, top) or covalently modified with QL47 (bottom). **e** HEK293T cells were transient transfected with FLAG tagged HSP70/HSC70 WT and C267S mutation plasmids for 48 h, cell lysis was incubated with gradient-diluted 47biotin, the combined HSP70 WT or C267S mutation were precipitated by streptavidin agarose and detected with FLAG antibody. **f** HSP70 ATPase activity was carried out with the co-chaperon protein HSP40 in presence of QL47 and VER155008 or DMSO for 1 h, the ADP produced were measured with ADP-Glo assay. Data represent a mean of triplicate ± SD. **g** In vivo firefly luciferase assay were carried out on HEK293T cells by transient-transfected pcDNA3.1-luciferase with pcDNA3.1-HSP70 or vehicle vectors. After 48 h, cells were heat shocked at 45 °C for 1 h, and then recovered at 37 °C for 0/0.5/1 h, luminescence in cell suspension were measured and presented as percentage of initial luminescence activity. With HSP70 overexpression, luciferase recovered to a higher level in 1 h, and the refolding of luciferase significantly slowed down upon 10 μM QL47 exposure in a time-dependent manner. Data represent a mean of triplicate ± SD. **h** Constitutive knockdown MOLM13/MV-4-11 cells were obtained by infection with lentivirus containing double-stranded shRNA hairpin DNA sequences targeting HSP70 or HSC70. The knockdown efficacy and the effect on FLT3 protein were detected by western blotting, using specific antibody. **i** The capacity of colony formation was detected in shSCR MOLM13, KD HSP70, and KD HSC70 MOLM13 cells. The colonies were counted in three fields, which selected randomly under the microscope, data represent a mean of triplicates ± SD. **j** MOLM13 cells with HSP70 or HSC70 knockdown were injected into nu/nu mice, five mice per group, in vivo tumor genesis, and growth ability were tested after cells inoculation for 14 days. The data are presented as a mean of tumor volume (mm^3^) in each group ± SD. **k** Analysis of HSP70 expression and survival of AML patients from the TCGA database by GEPIA. Low HSPA1B but not HSPA8 is highly related to the longer survival of AML patients. **l** Cell viability was measured by CellTiter-Glo assay after 72 h treatments with DMSO or gradient-diluted QL47 in two FLT3-ITD-positive AML patients’ derived cells P1 and P2. **m** FLT3-ITD proteins were degraded and apoptosis was induced by 12 h treatment of QL47 in P1 patient cells. **n** Antitumor efficacy of QL47 in a bone marrow engrafted mouse model using MV-4-11 cells. Survival curves are shown. **o** Body weight monitoring in the MV-4-11 cell bone marrow engrafted tumor model. *P value < 0.05, **P value < 0.01, ***P value < 0.001, and ****P < 0.0001 by unpaired *t* test

Unexpectedly, we observed that treatment with QL47 resulted in a decrease in FLT3-ITD protein in MV-4-11/MOLM13 cells in a time- and dose-dependent manner (Fig. 1b), and suppressed FLT3-STAT5-MYC signaling pathway activation (Supplementary Fig. S1b). Moreover, we found that QL47 induced a dramatic decrease in FLT3 proteins in transgenic BaF3 cells carrying the reported drug resistance mutations in the tyrosine kinase domain (TKD), such as F691L, N676D, and D835Y (Supplementary Fig. S1c), indicating that QL47 has potential for overcoming FLT3 drug resistance caused by secondary TKD mutations.

Using real-time RT-PCR, we first excluded the possibility that QL47 affects FLT3 through transcriptional regulation (Supplementary Fig. S1d). The Yang group reported that QL47 inhibits eukaryotic translation^3^, and we then examined QL47’s effects in the presence of the protein synthesis inhibitor cycloheximide (CHX) and proteasome inhibitor, MG132. As the results showed, QL47 in combination with CHX induced a more rapid decrease in FLT3-ITD protein than CHX alone, an effect enhanced by MG132 (Supplementary Fig. S1e). These results suggested that QL47, in addition to translation inhibition, also induced FLT3-ITD protein degradation through the proteasome pathway. With proteasome function blocked by MG132, unstable FLT3-ITD protein further aggregated into insoluble formations, as indicated by the increase in FLT3 protein we observed in the detergent-insoluble fraction (Supplementary Fig. S1e).

Heat shock proteins (HSPs) play important roles in protein homeostasis and folding, especially for the overexpressed or mutated oncoproteins in cancers. Therefore, we investigated the possible interactions between QL47 and HSPs, using a biotinylated QL47 derivative, 47biotin^2^. We unexpectedly found that HSP70, but not HSP90, precipitated with streptavidin agarose in a competitive manner (Fig. 1c), even under stringent high-salt conditions, suggesting a possible covalent interaction of QL47 and HSP70 (Supplementary Fig. S1f).

Then, we validated the binding between HSP70 and QL47 by mass spectrometry analysis and found that the TAC^267^ER peptide in HSP70 was modified upon interacting with QL47 (Fig. 1d), confirming a covalent bond between HSP70 and QL47. Consistently, the cysteine-to-serine mutation (Cys267Ser) of both inducible HSP70 and constitutive HSC70 disrupted the interaction of HSP70 and 47biotin, compared to that in cells overexpressing wild-type proteins (Fig. 1e). To further confirm the binding site of QL47, truncated proteins, namely the HSP70 nucleotide-binding domain (NBD; a.a. 1–382) and substrate-binding domain (SBD; a.a. 383–642), were transfected into HEK293T cells and precipitated with 47biotin, the results showed that the NBD but not the SBD retained the interaction with 47biotin, indicating that QL47 indeed targets the HSP70 protein at the NBD (Supplementary Fig. S1g).

ATPase and protein refolding assays showed that QL47 has a distinct action mode compared to the canonical ATP-competitive HSP70 inhibitor, VER155008. QL47 inhibited luciferase refolding by HSP70, but had no effect on the ATPase activity of HSP70 (Fig. 1f, g). Consistently, the computer-aided structure analysis showed that QL47 binds to HSP70 in a large groove near the ATP-binding pocket without occupying the ATP-binding site (Supplementary Fig. S2a). Upon QL47 binding, Cys267 located at the large groove is exposed to the solvent and reacts with the acrylamide of QL47 to form a covalent bond (Supplementary Fig. S2b), which explains why QL47 did not inhibit ATPase activity.

As a chaperone protein, HSP70 maintains cancer cell survival through multiple survival pathways. However, the effect of HSP70 on FLT3-ITD has not been reported. To confirm the role of HSP70 in FLT3-ITD-positive AML, we first validated the interaction between HSP70 and FLT3 proteins by co-immunoprecipitation, and the results revealed that endogenous HSP70 precipitated with FLT3-ITD proteins overexpressed in HEK293T cells (Supplementary Fig. S3a). Treatment of VER155008 in MV-4-11/ MOLM13 cells induced FLT3 destabilization and suppressed FLT3-STAT5-MYC signaling pathway (Supplementary Fig. S3b). In addition, we found that knockdown of inducible HSP70, but not constitutive expressed HSC70 by shRNAs was accompanied by more significate decreased FLT3 protein levels (Fig. 1h and Supplementary Fig. S4a) and cell viability in MV-4-11/MOLM13 cells (Supplementary Fig. S4b). Furthermore, feedback stimulation of HSP70 transcription in response to HSC70 knockdown was consistent with a previous report (Supplementary Fig. S4c)^4^. Moreover, HSP70 knockdown reduced the colony formation and tumor formation capabilities of MOLM13 cells in vivo more dramatically than HSC70 knockdown (Fig. 1i, j and Supplementary Fig. S4d, e). Previous research showed that the knockdown of either HSC70 or HSP70 alone has no effect on cell proliferation or client protein folding; only the dual inhibition of HSP70 and HSC70 has an effect^4^. However, we showed here that in FLT3-ITD-positive AML cells, knockdown of inducible HSP70 alone was sufficient to destabilize FLT3-ITD protein and inhibit the cell proliferation. In addition, TCGA (The Cancer Genomic Atlas) database analysis revealed that high expression of HSP70 (HSPA1B), but not HSC70 (HSPA8), was associated with poor AML survival (Fig. 1k). All these results suggest that HSP70 plays a more important role than HSC70 in the progression of FLT3-ITD-positive AML.

We next examined the antileukemic efficacy of QL47 in FLT3-ITD-positive patient-derived cells (Supplementary Table S1) and found that QL47 inhibited the proliferation of these cells (Fig. 1l). As HSP70 also functions independently of HSP90 to regulate apoptosis, we observed that QL47 also induced apoptosis, as evidenced by PARP, caspase-3 cleavage, and a decrease in X-linked inhibitor of apoptosis protein (XIAP), a client protein of HSP70 (Fig. 1m). Moreover, in midostaurin-resistant MV-4-11 cells (with N676D mutation), QL47 showed more potent anti-proliferation activity than midostaurin (Supplementary Fig. S5a), induced apoptosis, and decrease of FLT3-ITD-N676D/MYC proteins (Supplementary Fig. S5b). In addition, QL47 sensitized MV-4-11-MR cells to midostaurin by sixfolds (Supplementary Fig. S5c).

Finally, the antitumor efficacy of QL47 was evaluated in MV-4-11 cell-driven mice bone marrow engraftment model, QL47 not only significantly extended the animal survivals, but also dramatically reduced the number of MV-4-11 cells in the bone marrow (Fig. 1n, o and Supplementary Fig. S6). And in subcutaneous xenograft model using MV-4-11 cells, at a dosage of 10 mg/kg/day, QL47 achieved a tumor growth inhibition effect of 76.64% (Supplementary Fig. S7a, b). In addition, FLT3 and MYC proteins decreased in tumors administered with QL47 (Supplementary. Fig. S7c).

In conclusion, our study discovered a novel HSP70 inhibitor with potent antitumor efficacy, and showed that targeting HSP70 might be a promising therapeutic approach for the treatment of FLT3-ITD-positive AML with the potential to overcome drug resistance. Since QL47 was also reported to have antiviral effects by translation initiation inhibition^3,5^, further investigation is necessary to determine whether the antitumor activity of QL47 is caused by the dual inhibition of HSP70 and translation.

## Supplementary information

SUPPLEMENTAL MATERIAL

## Data Availability

The data that support the findings of this study are available from the lead corresponding author upon reasonable request.
