# Supplementary material for: Targeting chaperon protein HSP70 as a novel therapeutic strategy for FLT3-ITD-positive acute myeloid leukemia
Source: Signal Transduct Target Ther. 2021 Sep 15;6:334. doi: 10.1038/s41392-021-00672-7 (PMC8440619; doi:10.1038/s41392-021-00672-7)
Supplement: Supplementary file 1 — SUPPLEMENTAL MATERIAL [file 41392_2021_672_MOESM1_ESM.docx]

Supplementary Materials for

**Targeting Chaperon Protein HSP70 as a Novel Therapeutic Strategy for FLT3-ITD Positive Acute Myeloid Leukemia**

Chen Hu^1,2,6^, Fengming Zou^1,2,6^, Aoli Wang^1,2,6^, Weili Miao^3^, Qianmao Liang^1,4^, Ellen L. Weisberg^5^, Yinsheng Wang^3^, Jing Liu^1,2^*, Wenchao Wang^1,2^*, Qingsong Liu^1,2,4^*

1. Anhui Province Key Laboratory of Medical Physics and Technology, Institute of Health and Medical Technology, Hefei Institutes of Physical Science, Chinese Academy of Sciences, Hefei, Anhui 230031, P. R. China
2. Hefei Cancer Hospital, Chinese Academy of Sciences, Hefei, Anhui 230031, P. R. China
3. Department of Chemistry, University of California-Riverside, Riverside, CA 92521, USA
4. University of Science and Technology of China, Hefei, Anhui 230036, P. R. China
5. Department of Medical Oncology, Dana Farber Cancer Institute, Harvard Medical School, Boston, MA 02115, USA
6. These authors contribute equally

**Corresponding Authors**

* Qingsong Liu (E-mail: [qsliu97@hmfl.ac.cn](mailto:qsliu97@hmfl.ac.cn))

* Wenchao Wang (Email: [wwcbox@hmfl.ac.cn](mailto:wwcbox@hmfl.ac.cn))

* Jing Liu (E-mail: [jingliu@hmfl.ac.cn](mailto:jingliu@hmfl.ac.cn))

**This PDF file includes:**

Materials and Methods

Figures. S1 to S7

Tables S1

**Materials and Methods**

**Cell culture and cell growth inhibition assay**

The human AML cancer cell lines MV4-11, MOLM13 and MOLM14 were provided by Dr. Scott Armstrong, Dana Farber Cancer Institute (DFCI), Boston, MA, USA. MV-4-11 cells were cultured with Iscove’s Modified Dulbecco’s Medium with 10% FBS, MOLM13 and MOLM14 were cultured with RPMI medium with 10% FBS (#FND500) (Excell, SuZhou, China). The Briefly, 3000 cells were seeded in 96-well plates and treated with serially diluted compounds for 72 h. Then, CellTiter Glo reagent (Promega, Madison, WI, USA) was added to evaluate antiproliferation activity.

**Antibodies and reagents**

Anti-Hsc70 antibody (#ab19136) were purchased from Abcam(Cambridge, MA, USA); FLT3 antibody (#3462), HSP70 antibody (#4873), Phospho-Stat5 (Tyr694) antibody (#4322),[c-Myc antibody](http://www.cst-c.com.cn/products/5605.html) (#5605), XIAP antibody (#14334), PARP antibody(#9532), Mcl-1 antibody(#94296) and Caspase-3 Antibody (#9665) were purchased from Cell Signaling Technology (Danvers, MA,USA). Anti-FLAG M2 Gels (#A2220) and anti-FLAG antibody (#F3165) were obtained from Sigma-Aldrich ([St. Louis, Missouri](https://cn.bing.com/search?q=St.+Louis,+Missouri+wikipedia&FORM=LFACTRE), United States). Streptavidin Agarose (#20347) was obtained from Thermo Fisher Scientific (Waltham, MA, USA).

**Chemical synthesis and characterization of QL47 and 47Biotin**

The QL47 and 47biotin were synthesised as previous reported^1^.

**Real-time RT-PCR**

Cells were collected, and total RNA was extracted by an RNAprep Pure Kit (TIANGEN, DP430). cDNA samples were prepared by reverse transcription with the Prime Script™ RT Reagent Kit (TAKARA, RR037), and mRNA levels were detected by real-time PCR with TB Green Premix Ex Taq II (TAKARA, RR820A). All experiments were performed in triplicate.

**PCR Primer used for real time PCR**

FLT3: TCTGCTTCCATCACACTGCAA

CTGGCAATTCAGGGAGCTGTG

HSP70: CATCTCGTGGCTGGACGC

CCTGAGCCCCGAAGCCC

HSC70: GGCTTGATAAGAATCAGACTGCTGA

CTCCACCACCAGGAAATCCC

GAPDH: TGGTCACCAGGGCTGCTTTTA

TTCCCGTTCTCAGCCTTGACG

**HSP70 ATPase assays**

Briefly, 0.1 μM HSP70 was incubated with the serially diluted compound for 30 min at room temperature; then, 0.1 µM HSP40/1 µM ATP mixtures were added and reacted at room temperature for 1 h. The ADP produced in the reaction was detected by the ADP-Glo assay from Promega. The luminescence was measured with a plate reader ENVISION, Perkin-Elmer (Waltham, MA, USA)^2^.

**In vivo luciferase refolding assay**

HSP70-pcDNA3.1 or empty vector was cotransfected with the luciferase-pcDNA3.1 plasmid into HEK293T cells for 48 h. Cells were treated with 50 μg/ml cycloheximide together with QL47 or DMSO and subjected to immediate heat shock at 45°C for 1 h. After recovery for 1 h at 37°C, cells were collected and lysed. Refolded luciferase activity in suspension was then measured by the Bright-Glo reagent (Promega) with a plate reader ENVISION and normalized by the protein concentration determined by the BCA assay^2^.

**DNA Transfection, Chemical and immunoprecipitation, Protein Extraction and Western Blot Analysis**

For immunoprecipitation, FLT3-ITD were overexpressed in HEK293T by transfection with pcDNA3.1-FLT3-ITD-FLAG vector; for chemical precipitation assays, HEK293T cells were transfected with pcDNA3.1 vectors containing full-length HSP70 wild-type, C267S mutant, HSP70 (1-382) or HSP70(383-642) truncations. For all transfections, HEK293T were seeded in plates for 12h before DNA were mixed with Lipofiter^TM^ transfection reagent (Hanbio, shanghai, China) in a ratio of 1:3. Cells were harvested 48 h after transfection for precipitation and Western blot.

Cells were lysed by RIPA (Beyotime #P0013C) supplemented with protease/ phosphatase Inhibitor Cocktail (Cell Signaling Technology #5872). The suspension of cell lysis was boiled with 5*loading buffer, 40ug proteins were load on SDS–PAGE and western blot, then detected with specific antibodies. The pellet after centrifuge was washed by PBS buffer and resuspended with pellet buffer as reported^3^.

HEK293T cells overexpressing FLAG-tagged FLT3-ITD were lysed and FLAG-tagged proteins were immunoprecipitated with anti-FLAG M2 Affinity Gel. For the biotin probe, cell lysates were treated with serially diluted QL47 followed by 10 uM 47biotin overnight and precipitated with Streptavidin agarose. The beads were then washed at least 3 times with different buffers and eluted with 2X loading buffer for SDS-PAGE and western blot.

**Primary cells treatments**

Primary cells from AML patients were obtained from the First Hospital of Anhui Medical University, Anhui, China. Peripheral blood and bone marrow mononuclear cells were isolated by the Ficoll-Paque method^4^. All studies performed with human specimens were performed with approval from the First Hospital of Anhui Medical University. Ethical approval and informed consent were obtained for the use of human samples.

**FLT3-BaF3 transgenic cell lines generation**

FLT3-BaF3 transgenic cell lines that expressed different FLT3 mutations were constructed as previous reported^5^.

**Stable cell line generation with HSP70 knockdown**

high-titer lentivirus with double-stranded shRNA hairpin DNA sequences targeting HSP70 or HSC70 genes were obtained from GenePharma (Shanghai). Stable knockdown cell lines were selected with 0.5 µg/ml puromycin for 48 h. Knockdown efficiency was evaluated by real time RT-PCR and Western blot using specific HSP70 and HSC70 antibodies.

shRNA used for knockdown HSP70 isoforms:

sh HSP70: GGACGAGUUUGAGCACAAG

sh HSC70: GCAAAGAAUCAAGUUGCAAUG

**Colony formation assay**

The bottom layer of six-well tissue culture dishes was composed of 1 mL of 3 % agarose and 1 mL cell culture medium. 3000 MOLM13 cells were plated on top of the bottom layer maintained in 1.7 mL growth media combined with 0.3 ml of 3% agarose solution. Cells were maintained in a humidified 5% CO2 incubator at 37°C for 15 days. The colonies were counted under 10x objective lens in three fields of view randomly selected.

**Protein purification**

Full-length HSP70 gene fragments were constructed in p-FASTBAC HTA vector (Invitrogen) and used for expression in SF9 cells. HSP40 gene was constructed in pET28a vector and used for expression in BL21 cells. Proteins were expressed and purified as previously reported^6^. Briefly, the [supernatant](http://dict.cn/supernatant) after sonication was loaded to Ni-NTA Column (QIAGEN, 1018244). Then the proteins were step eluted with buffer containing 250mM imidazole. The eluted proteins were loaded on a Superdex-200 column equilibrated in 25mM Tris (pH 7.4), 250mM NaCl, 1mM DTT, and 1mM EDTA. Peak fractions were concentrated and used for ATPase assay.

**Mass Spectrometry Analysis**

Recombinant human HSP70 protein (at a final concentration of 10 µM) was incubated with QL47 (at a final concentration of 100 µM) in a buffer containing 50 mM HEPES (pH 7.4), 75 mM NaCl, and 5% glycerol at room temperature with gentle shaking for 2 h. After the reaction, the remaining QL47 and the reaction buffer were removed by exchange into a 50 mM NH_4_HCO_3_ (pH 8.5) solution with the use of a Microcon centrifugal filter (30,000 NMWL, Millipore). To the protein sample were subsequently added 8 M urea for protein denaturation, dithiothreitol and iodoacetamide for cysteine reduction and alkylation, respectively. The protein was then subjected to digestion with modified MS-grade trypsin (Thermo Pierce) at an enzyme/substrate ratio of 1:100 in 50 mM NH_4_HCO_3_ (pH 8.5) at 37 °C for overnight. The ensuing tryptic peptides were subsequently dried in a Speed-vac, desalted by using an OMIX C18 pipet tip (Agilent Technologies, Santa Clara, CA), and analyzed by LC-MS and MS/MS.

The LC-MS and MS/MS experiments were conducted on a Q Exactive Plus Quadrupole-Orbitrap mass spectrometer. The samples were automatically loaded from a 48-well microplate autosampler using an EASY-nLC 1200 system (Thermo Fisher Scientific) at 3 µL/min onto a biphasic precolumn (150 µm i.d.) comprised of a 3.5-cm column packed with C18 reversed-phase material (5 µm in particle size and 120 Å in pore size, ReproSil-Pur 120 C18-AQ, Dr. Maisch). The biphasic trapping column was connected to a 20-cm fused-silica analytical column (PicoTip Emitter, New Objective, 75 µm i.d.) packed with 3 µm C18 beads (ReproSil-Pur 120 C18-AQ, Dr. Maisch). The peptides were then separated using a 180-min linear gradient of 2-45% acetonitrile in 0.1% formic acid and at a flow rate of 300 nL/min. The mass spectrometer was operated in a data-dependent scan mode. Full-scan mass spectra were acquired in the range of *m/z* 300-1500 in the Orbitrap analyzer with a resolution of 70,000. Up to 25 most abundant ions found in MS with a charge state of 2 or above were sequentially isolated and collisionally activated in the HCD cell with collision energy of 28 to yield MS/MS.

**Mice models**

All animals were purchased from Beijing Vital River Laboratory Animal Technology Co., Ltd (Beijing, China), housed in a specific pathogen-free facility and used according to the animal care regulations of Hefei Institutes of Physical Science Chinese Academy of Sciences (Approval no. HFCASDWLL 20160320).

For xenograft tumor model, four-week old female nu/nu mice AML cells were formulated as a 1:1 mixture with Matrigel (BD Biosciences) (San Jose, CA, USA) and subcutaneously injected into the right flank of nu/nu mice. After inoculation, tumor length(L) and width(W) were measured every 2 days to calculate tumor volume (＝[(W^2^ × L)/2]). QL47 was delivered daily in HKI solution (0.5% methylcellulose/0.4% Tween 80 in ddH2O) by intraperitoneal injection.

For engraftment tumor model, five-week-old female NOD-SCID mice were intraperitoneally injected with cyclophosphamide (CTX) 50mg/kg daily for two days. 24 hours later, 8 million MV-4-11 cells in 0.3 mL IDMEM medium were injected through intravenous administration (tail vein). 3 weeks after cell inoculation, 5 mg/kg QL47 and 50mg/kg midostaurin were initiated daily (intraperitoneal administration). Mice were monitored daily and were euthanized when moribund or at early signs of hind limb paralysis. After the experiment, the cells in bone marrow were isolated and analyzed by flow cytometry with PE conjugated HLA-ABC (G46e2.6) and evaluated by FlowJo software.

**Figure S1**

**
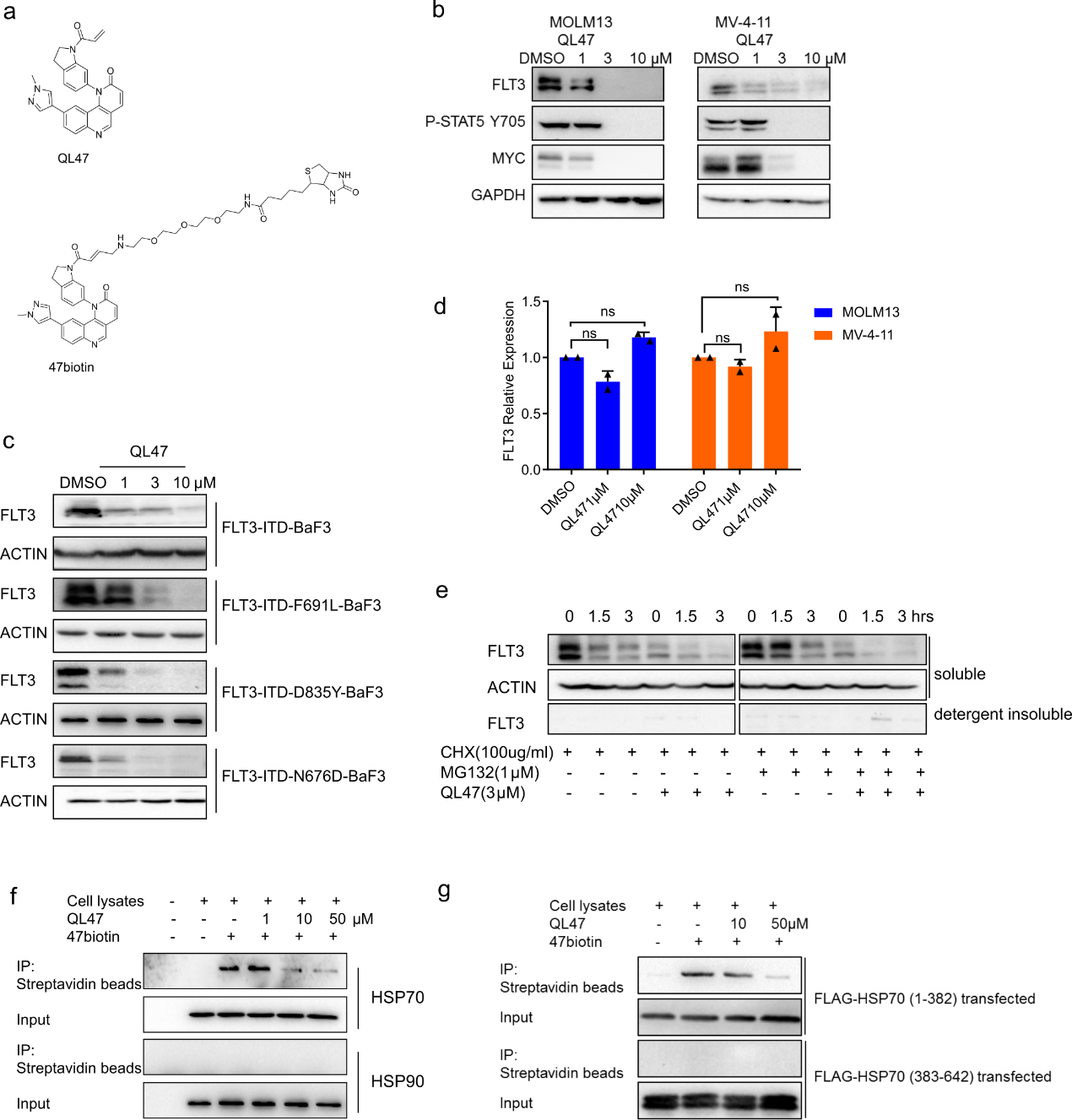
**

## Supplementary Figure S1 a The chemical structure of QL47 and 47biotin. b QL47 suppressed the FLT3-STAT5-MYC signal pathway in MOLM13 and MV-4-11 cell lines. c QL47 induced degradation of FLT3-ITD proteins containing resistance mutations in transgenic FLT3-mutant-BaF3 cell lines. d Real time RT PCR analysis for the FLT3 transcription level in MV-4-11 and MOLM13 cell lines with QL47 treatment for 6 h. e MOLM13 cells were treated with 3μM QL47 for 3h, followed by 1μM MG132 treatment for 1h, then exposure to 100ug/ml CHX, cells were collected at 0/1.5/3 h. The FLT3 in detergent soluble and insoluble particles were tested by western-blot. f QL47 preincubated MV-4-11 cell lysate were competed with 47biotin, then precipitated by streptavidin agarose and washed by 20mM Tris pH7.5,1M NaCl 1%NP40 buffer, heat shock proteins binding with 47biotin were detected by Western-blot using HSP70, HSP90 antibodies. g HSP70 truncations NBD domain (1-382) and SBD domain (382-642) were overexpressed in HEK293T cells, cell lysis were incubated with QL47 followed by 47biotin, and precipitated by streptavidin agarose. The combined domain of HSP70 was detected by western-blot and FLAG antibody.

## Figure S2


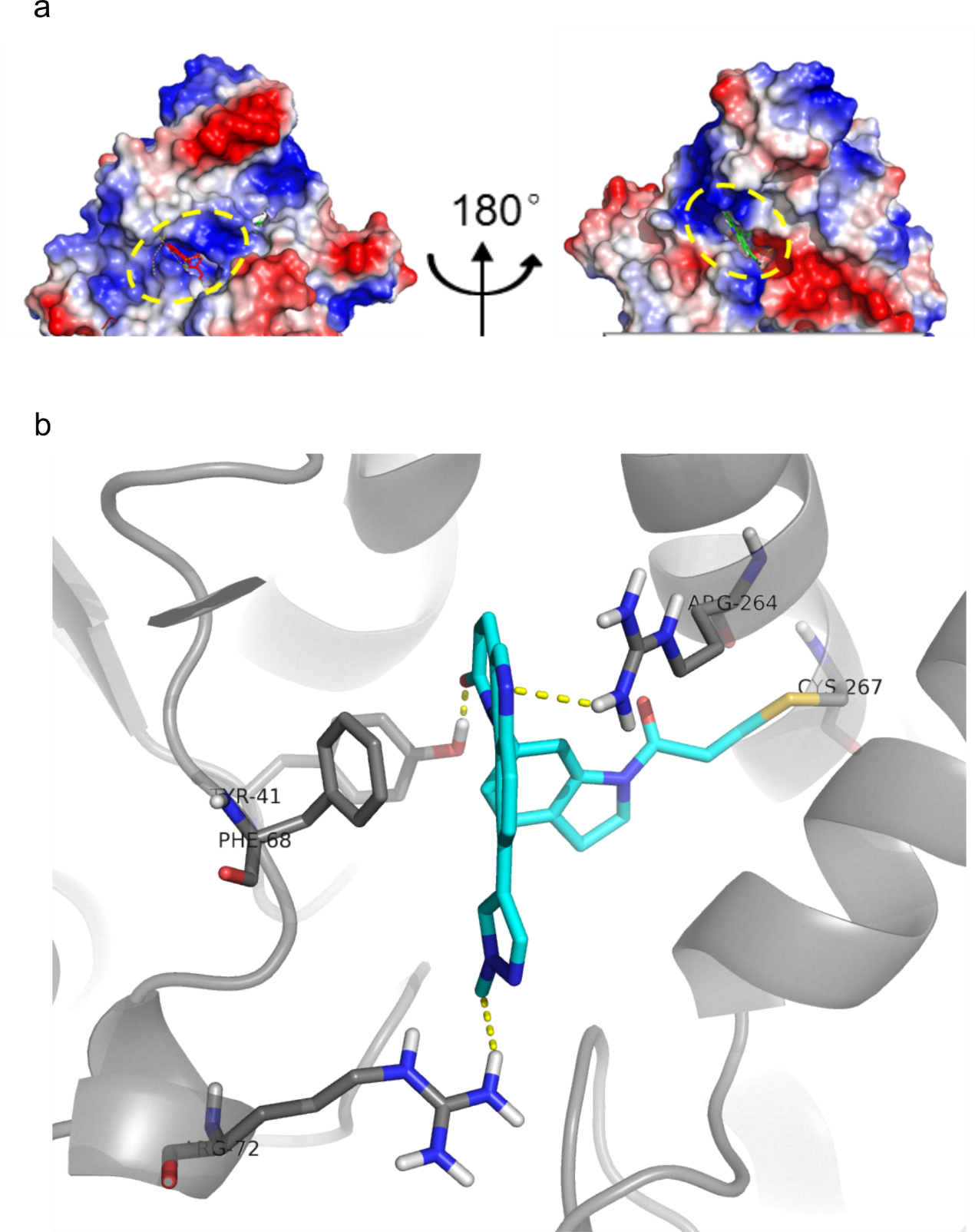


## Supplementary Figure S2 a QL47-HSP70 binding mode were modeled based on the HSP70 structure (PDB ID: 5AQW) by Covdock software. Structural diagram of the HSP70 highlighting the ATP-binding site and QL47 binding site (Yellow dotted line). ADP (Red) and QL47 (Green) are shown bound to each site, respectively (PDB: 5AQW). b Irreversible bond was formed between Cys267 and acrylamide of QL47, hydrogen bonds between QL47 and HSP70 were illustrated as yellow dotted lines.

## Figure S3

**
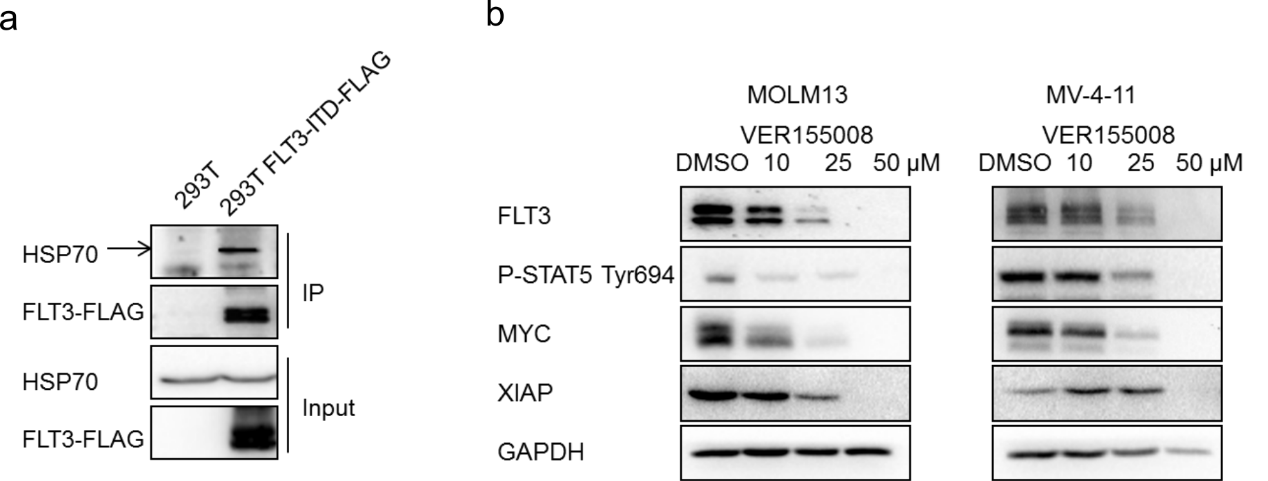
**

**Supplementary Figure S3 a** FLAG-tagged FLT3 proteins were overexpressed by transient transfection FLT3-ITD-pcDNA3.1 vector in HEK293T for 48 h, endogenous HSP70 was co-immunoprecipitated with anti-FLAG affinity gel. **b** The effect of VER155008 on FLT3-STAT5-MYC signal pathway was investigated in MOLM13 and MV-4-11 cells.

## Figure S4


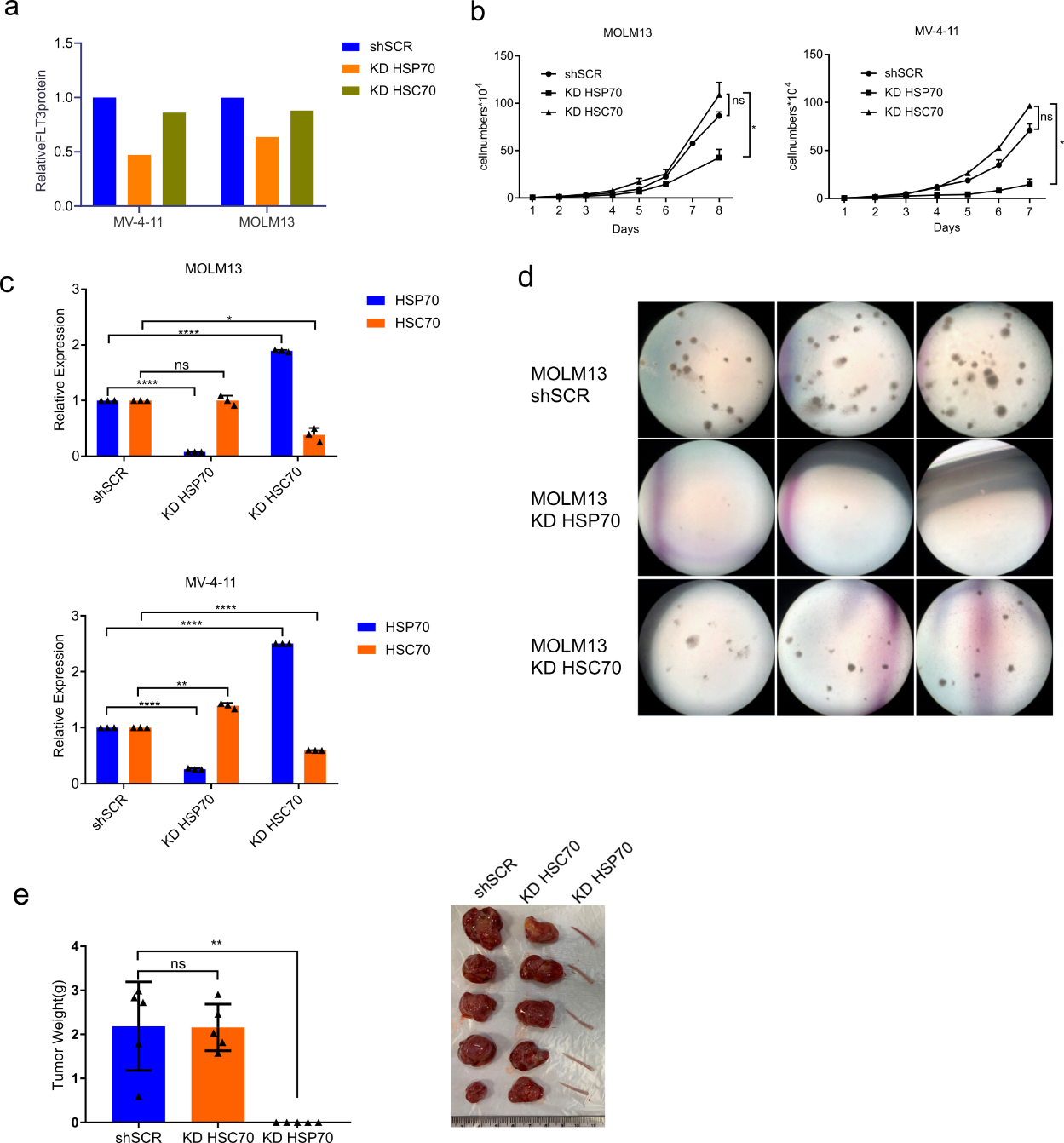


## Supplementary Figure S4 a The FLT3 protein levels in shSCR , KD HSP70, and KD HSC70 MOLM13/MV-4-11 cells were quantified by ImageJ software. b Proliferation curve of shSCR , KD HSP70, and KD HSC70 MOLM13/MV-4-11 cells were measured for 8 days. Data represent a mean of duplicate ±SD. c The gene expression level of HSP70 and HSC70 were validated by Real time RT-PCR in HSP70 or HSC70 knockdown MV-4-11/MOLM13 cells. Data represent a mean of triplicates ±SD and as fold changes compared with the MV-4-11 and MOLM13 cells infected with scramble shRNA. d In colony formation assay, the colonies were counted in three fields which selected randomly under the microscope. Photographs of each field are shown here. e Left picture presented the tumor weight in each group after HSC70 or HSP70 knockdown in MOLM13 cells as a mean ±SD. Right picture are representative photographs of tumors in each group. **P*-value < 0.05, ***P*-value < 0.01, ****P*-value < 0.001, and *****P* < 0.0001 by unpaired *t* test.

## Figure S5

##
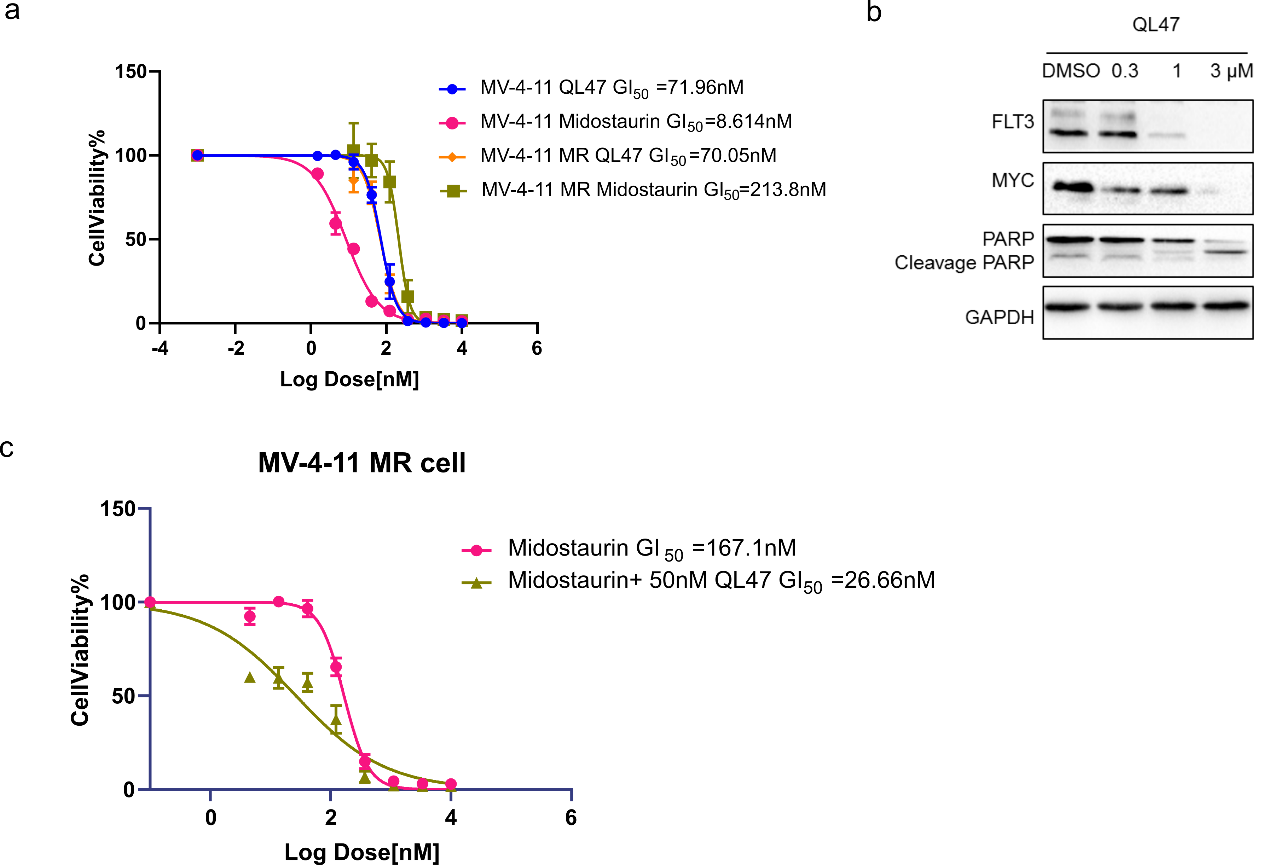


## Supplementary Figure S5 a Anti-proliferation activity assay of QL47 and midostaurin in MV-4-11 and MV-4-11-MR (midostaurin-resistant) were performed after 72-hour treatment. b Effects of QL47 on FLT3-ITD-N676D, MYC, and apoptosis in MV-4-11-MR cells were investigated by Western Blot after 24-hour treatment. c QL47 (50 nM) enhanced the sensitivity in MV-4-11-MR cells to midostaurin. Cell viabilities were evaluated by CellTiter-Glo assay after 72 hours of treatment.

## Figure S6

##
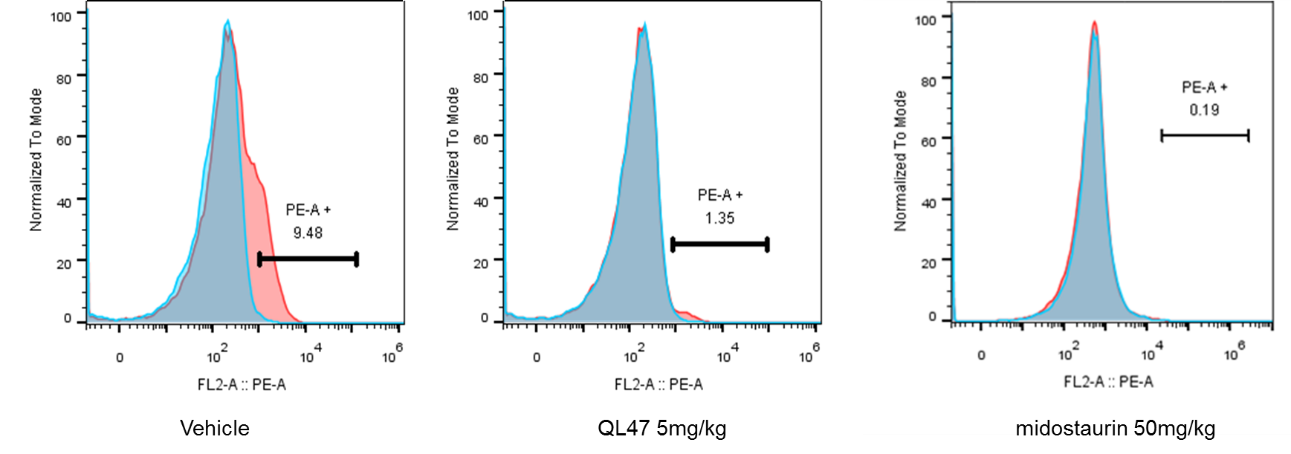


## Supplementary Figure S6 In the bone marrow engrafted mouse model, 5mg/kg dosage of QL47 reduce the MV-4-11 cells in the bone marrow significantly.

## Figure S7


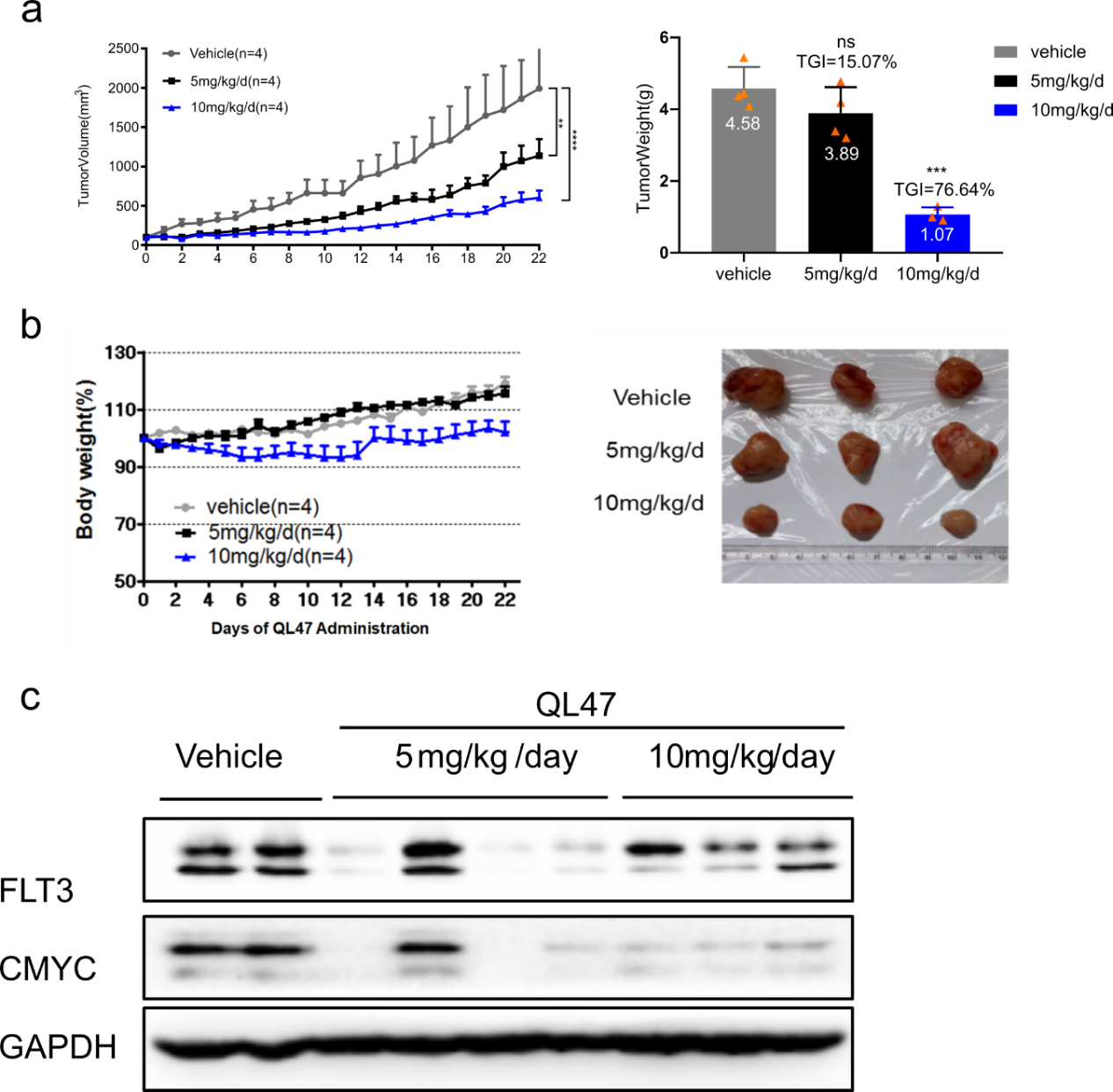


## Supplementary Figure S7 a QL47 efficacy was evaluated in MV-4-11 inoculated xenograft mouse tumor model. Tumor volumes in each group (n=4) were measured for 22 days. The tumor weights were detected after 22 days and QL47 exhibited potent anti-tumor efficacy with tumor growth inhibition (TGI) of 76.64% by 10mg/kg/day treatment. b Mice body weight measurement (Left) and representative photographs of tumors in each group after 5, 10mg/kg/d QL47 or vehicle treatment in MV-4-11 xenograft models (Right). The FLT3 and MYC protein levels in tumor tissues were also decreased which detected by western-blot (c).

**Supplementary Table S1: Patient primary cell information**

| **Samples** | **Age** | **Gender** | **Pathology** | **Blast**  **(%)** | **Cytogenetics** |
| --- | --- | --- | --- | --- | --- |
| P1 | 73 | F | AML-M5 | 82.5 | 62.7K WBC count;  previous therapy:  idarubicin, high dose cytarabline, mitoxantrone; cytogenetics: normal; mutations:  dupMLL+; FLT3-ITD (17 AA,  FLEYEYDLKWEFPRENL) |
| P2 | 55 | M | AML-M5 | 49 | 226.03K WBC count;  previous therapy：  homoharringtonine, cytarabine and recombinant hHuman granulocyte colony-stimulating factor;  cytogenetics:normal;  mutations:  FLT3-ITD(7AA, FREYEYD) |

1 Wu, H. *et al.* Discovery of a potent, covalent BTK inhibitor for B-cell lymphoma. *ACS Chem Biol* **9**, 1086-1091 (2014). doi:10.1021/cb4008524

2 Rodina, A. *et al.* Identification of an allosteric pocket on human hsp70 reveals a mode of inhibition of this therapeutically important protein. *Chem Biol* **20**, 1469-1480 (2013). doi:10.1016/j.chembiol.2013.10.008

3 Guo, L., Prall, W. & Yang, X. Assays for the Degradation of Misfolded Proteins in Cells. *J Vis Exp* (2016). doi:10.3791/54266

4 Wottawa, A., Klein, G. & Altmann, H. [A method for the isolation of human and animal lymphocytes with Ficoll-Urografin (author's transl)]. *Wien Klin Wochenschr* **86**, 161-163 (1974).

5 Wu, H. *et al.* Discovery of a highly potent FLT3 kinase inhibitor for FLT3-ITD-positive AML. *Leukemia* **30**, 2112-2116 (2016). doi:10.1038/leu.2016.151

6 Bhangoo, M. K. *et al.* Multiple 40-kDa heat-shock protein chaperones function in Tom70-dependent mitochondrial import. *Mol Biol Cell* **18**, 3414-3428 (2007). doi:10.1091/mbc.e07-01-0088
